# Heterochrony repolarized: a phylogenetic analysis of developmental timing in plethodontid salamanders

**DOI:** 10.1186/2041-9139-5-27

**Published:** 2014-08-18

**Authors:** Ronald M Bonett, Michael A Steffen, Grant A Robison

**Affiliations:** 1 Department of Biological Science, University of Tulsa, Tulsa, OK 74104, USA

**Keywords:** Ancestral state reconstruction, Caudata, Evolution, Life history, Neoteny, Paedomorphosis, Progenesis

## Abstract

**Background:**

Disentangling evolutionary shifts in developmental timing (heterochony) is dependent upon accurate estimates of ancestral patterns. However, many classic assessments of heterochronic patterns predate robust phylogenetic hypotheses and methods for trait reconstruction, and therefore may have been polarized with untested ‘primitive’ conditions. Here we revisit the heterochronic modes of development that underlie the evolution of metamorphosis, maturation, and paedomorphosis in plethodontid salamanders. We focus on the tribe Spelerpini, which is a diverse clade that exhibits tremendous variation in timing of metamorphosis and maturation, as well as multiple independent instances of larval form paedomorphosis. Based on morphology and biogeography, early investigators concluded that the most recent common ancestors of plethodontids, and also spelerpines, were large salamanders, with very long larval periods and late maturation times. This prevailing assumption influenced subsequent heterochronic assessments, which concluded that most modern spelerpines (with shorter larval periods) were derived through multiple independent accelerations in larval development. It was also concluded that most occurrences of larval form paedomorphosis in this clade resulted from progenesis (acceleration of gonadal development relative to metamorphosis).

**Results:**

By reconstructing the time to metamorphosis on a molecular-based phylogeny of plethodontids, we find that ancestral spelerpines likely had relatively shorter larval periods than previously proposed. Taken together with the credibility interval from our ancestral state estimation we show that very long larval periods are likely derived decelerations, only a few lineages have undergone appreciable accelerations in metamorphic timing, and the remaining taxa have lower probabilities of being different than the ancestral condition (possibly due to stasis). Reconstructing maturation age across nodes concomitant with the evolution of larval form paedomorphosis in one large radiation does not show clear evidence of progenesis, but more likely indicates a case of neoteny (delayed metamorphosis).

**Conclusions:**

This study demonstrates cases in plethodontid salamanders where phylogenetic-based character reconstructions reject previously hypothesized ancestral life history conditions. As a result, several prior hypotheses of heterochronic evolution in this family are reversed.

## Background

Shifting the ontogenetic time of developmental events may be a primary mechanism for producing diversity of morphology and life history [1-6]. Disentangling specific heterochronic patterns can provide insight into the direction, developmental mechanisms, and ecological circumstances that promote evolutionary shifts [1-10]. However, conducting comparative analyses of heterochronic patterns can be challenging and easily confounded. This is because these analyses rely on knowledge of the timing of developmental events for multiple taxa, an understanding of their phylogenetic relationships, and methods to deduce ancestral patterns of development [11-19]. Evolutionary and developmental biologists have been interested in heterochronic patterns for several decades, with the greatest surge of papers over the last 35 years [1,2,20]. However, only recently have robust phylogenies of many clades come into focus, as well as adequate methods for reconstructing and testing ancestral states. This suggests that some non-phylogenetically (or non-paleontologically) polarized heterochronic assessments may need to be reconsidered in light of more refined phylogenetic hypotheses and/or ancestral state reconstruction methods.

**Figure 1:**
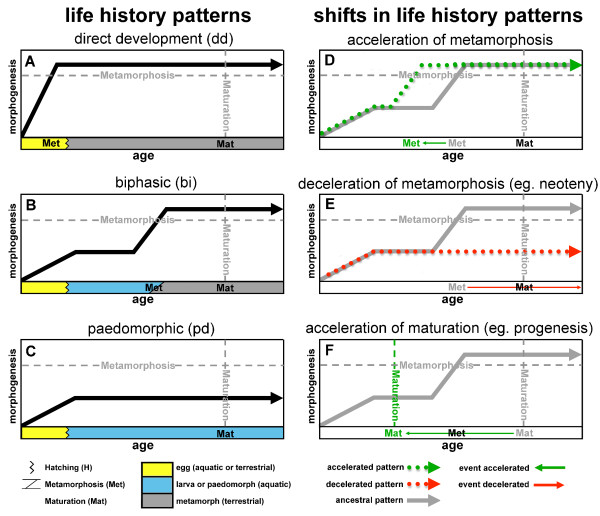
**Developmental timing and life history events of plethodontids.** The solid black line represents morphogenic changes for three alternative life history strategies **(A-C)**: direct development, biphasic, and paedomorphic. Dashed horizontal and vertical grey lines indicate metamorphosis and maturation, respectively. **(A)** Direct developing plethodontids metamorphose inside of the egg, usually followed by a terrestrial juvenile stage and terrestrial maturation. **(B)** Biphasic plethodontids typically have aquatic eggs, aquatic larval stages (of varying durations), and metamorphose into a more terrestrial morphology prior to, or around the time of, maturation. **(C)** Paedomorphs mature while still in the larval form prior to metamorphosis, which may, or may not, subsequently occur. Accelerations and decelerations in somatic morphogenesis and maturation can alter the timing of life history events **(D-F)**. The solid grey lines represent an example of ancestral morphogenic changes, and in these examples the ancestral life history is biphasic (the same as B). **(D)** Dotted green line shows a trajectory with an accelerated timing of metamorphosis compared to its ancestor. In this case, the acceleration still results in a biphasic descendent, but with an abbreviated larval period. Further acceleration of morphogenic changes (completed within the egg) could lead to direct development. **(E)** Dotted red line shows a trajectory with decelerated timing (or permanent postdisplacement) of metamorphosis compared to its biphasic ancestor. This example of deceleration shows neoteny, where the deceleration results in maintenance of the larval form beyond maturation (paedomorphosis). **(F)** Dashed green vertical line represents an acceleration in the timing (or predisplacement) of maturation to an age prior to metamorphosis, resulting in a progenic descendant exhibiting larval form paedomorphosis. It is important to note that accelerations (and decelerations) in maturation time also occur within life history categories. Terminology is from [26] and the figure is a synthesis from [7,23,26,27] with modifications and additions.

The phenomenon of ‘larval form paedomorphosis’ in salamanders has been a hallmark example of heterochrony [1]. This developmental pattern occurs when adults retain suits of larval juvenile characteristics and aquatic ecology into adulthood, and has independently evolved multiple times in salamanders [21-25]. There are at least two general ways in which larval form paedomorphosis can occur: (1) neoteny, which is decelerating or delaying metamorphic changes (somatic morphogenesis) despite consistent timing `of reproductive development compared to ancestors; and (2) progenesis, which is accelerating or advancing gonadal development relative to somatic development. In other words, maturation precedes metamorphosis, resulting in an adult with a larval morphology (Figure 1; see also Methods section for additional qualifications of terminology). Quantitative tests of whether neoteny or progenesis is responsible for paedomorphosis in salamanders have been restricted to a few intraspecific comparisons in facultatively paedomorphic species [7,8]. However, heterochronic analyses of metamorphosis and maturation have not been conducted in a phylogenetic context to test the developmental origins of larval form paedomorphosis for any salamander clades.

The Family Plethodontidae is the most species rich clade of salamanders [21] and exhibits a wide range of life history strategies [21-26,28]. These include aquatic larvae followed by metamorphosis (biphasic), larval form paedomorphosis, and the absence of free-living aquatic larval forms (direct development; Figure 1). The biphasic life history occurs in at least three lineages of plethodontids (desmognathines, spelerpines, and the monotypic genus *Hemidactylium*). Given that most salamander families also have aquatic larvae, the first assessments of plethodontid evolution concluded that clades with biphasic taxa diverged early in the evolution of the family, and biphasic life history was the ancestral condition [29-31]. More specifically, based on morphology and biogeography, it was proposed that the ancestral ‘hemidactyline’ (spelerpines + *Hemidactylium*) was a large salamander, with a long larval period and late maturation time (similar to the genus *Gyrinophilus*[29-33]). Based on this hypothesis for the ancestral condition, subsequent estimates of heterochronic patterns in the ‘hemidactylines’ concluded that the shorter larval periods of most species groups evolved from multiple independent accelerations in larval development [26] (Figure 1).

This study [26] also concluded that most instances of larval form paedomorphosis in spelerpines (particularly the genus *Eurycea*) resulted from progenesis rather than neoteny (Figure 1). Since then, robust molecular phylogenies of plethodontids, have completely inverted our understanding of the directionality of life history evolution in this family with regard to direct development and the evolution of larval periods [28,34,35]. Furthermore, a recent phylogeny of spelerpines [25] has provided a framework for re-evaluating the modes of heterochrony in this clade, which includes all paedomorphic plethodontids. However, these studies analyzed the evolution of life history as a dichotomous trait (biphasic *vs.* direct development [28]; biphasic *vs.* paedomorphic [25]), and patterns of timing of metamorphosis and maturation have yet to be revisited.

Here we analyze patterns of heterochrony in plethodontids, by reconstructing ancestral larval periods and maturation times on a well-rooted phylogeny, and testing the fit of alternative ancestral states at key nodes. We use the credibility interval from continuous ancestral state estimation to further analyze patterns of acceleration and deceleration in a diverse clade (tribe Spelerpini). Furthermore, we re-evaluate the heterochronic patterns (neoteny *vs.* progenesis) that likely led to paedomorphosis in one large radiation of paedomorphic spelerpines from the Edwards Plateau of Central Texas. We show that, in light of the molecular phylogeny and ancestral state estimates, many previously hypothesized patterns of heterochrony in plethodontids are reversed. This study highlights the importance of ancestral state reconstruction and estimation limits for understanding patterns of heterochrony.

## Methods

### Terminology

We acknowledge that the history of heterochronic terminology has been tumultuous [1,2,27]. For comparative purposes, here we follow the terminology of Ryan and Bruce [26], which has been the only prior comprehensive treatment of heterochrony in spelerpine plethodontids. We use the terms acceleration and deceleration, respectively, to refer to the relative advancement and delay of the timing of developmental events compared to ancestors (Figure 1). These terms are applied to processes effecting somatic and reproductive tissues, which may, or may not, result in a shift between life history categories (direct development, biphasic, paedomorphic). For example, if metamorphosis of a biphasic species occurs significantly earlier than metamorphosis of its biphasic ancestor then this would be an acceleration in the age (timing) of metamorphosis.

Also following Ryan and Bruce [26] and other studies [8], we use the terms neoteny and progenesis primarily to refer to somatic deceleration and reproductive acceleration, respectively, which are processes that can result in larval form paedomorphosis. There are multiple ontogenetic trajectories that can lead to an advancement or delay of a developmental event. Since shifts in maturation and metamorphosis could be considered changes to the ‘onset’ or ‘offset’ of a developmental trajectory, then terms implying a ‘rate’ (neoteny, progenesis, acceleration, deceleration) may not apply. Instead, terms such as predisplacement and postdisplacement have been used to describe such shifts in the timing of reproduction and metamorphosis [2,7].

### Data

Data on timing of metamorphosis (age at metamorphosis) and maturation (age of gonadal maturation) in months for 63 plethodontids were primarily derived from the literature and some personal and unpublished observations by colleagues (Additional file 1). This sampling included representatives of most of the major lineages of plethodontids: 26 species of spelerpines from four of the five genera (21 *Eurycea*, 2 *Gyrinophilus*, 2 *Pseudotriton*, and 1 *Stereochilus*), 14 species of desmognathines (*Desmognathus*), and 23 other plethodontids (2 *Aneides*, 1 *Batrachoseps*, 2 *Bolitoglossa*, 1 *Ensatina*, 1 *Hemidactylium*, 1 *Hydromantes*, 14 *Plethodon*, and 1 *Pseudoeurycea*). Our analyses were based on minimum estimates of age at metamorphosis. Direct developing species metamorphose prior to hatching, so we considered their age at metamorphosis to be the time prior to hatching, which for most species was approximately 2 months [21]. Most paedomorphic plethodontids do not metamorphose (obligately paedomorphic), and this is an independently derived state in multiple lineages of spelerpines [25]. Since we were most interested in reconstructing the ancestral timing of metamorphosis, we coded paedomorphic taxa as missing metamorphic data in our analyses of metamorphic timing. We dealt with the evolution of larval form paedomorphosis (compared to direct development or biphasic) in a separate analysis (described below).

We also used minimum age estimates for maturation for all 63 species, and we analyzed male and female maturation times separately. We used minimum age (as opposed to average or maximum age) because it is the most consistent and obtainable metric across species. Most referenced studies are based on evaluating gonadal development across age/size classes. Therefore, we used minimum age at gonadal maturation (which is observed morphologically), as opposed to age at first reproduction (oviposition or spermatophore drop), which are less commonly documented. For example, the minimum age of reproductive maturation for both male and female *Desmognathus ocoee* has been documented at 3 years [36,37]. Even though most female *D. ocoee* may not oviposit until year 4, we used 3 years to be consistent with other studies that are only based on gonadal maturation.

There have been phylogenetic based reconstructions of plethodontid life history: biphasic *vs.* direct development [28] and biphasic (metamorphic) *vs.* paedomophic [25]. However, these three states have not been reconstructed in the same analysis. Therefore, we also reconstructed ancestral life history (direct development, biphasic, paedomorphic) for 100 plethodontids, including all North American and Eurasian genera, as well as a newly described paedomorphic species (*E. subfluvicola*[38]). Life history information for these species is well established and was taken from the literature (Additional file 1). We only included three representative genera from the tropical radiation (bolitoglossines), due to the limited number of lengthy *Rag1* sequences available for this group (see below), but it is clear that this radiation is monophyletic, and all species are thought to be direct developers. In other words, including additional bolitoglossines to our analyses would not significantly change the results presented here. The purpose of this analysis was primarily to reconstruct the origins of paedomorphosis within spelerpines, which was necessary for subsequent tests of progenesis *vs.* neoteny (see below). However, we also performed additional life history reconstructions and included the outgroup families Amphiumidae and Rhyacotritonidae to further test the ancestral life history mode of plethodontids (see Results).

### Phylogeny

We reconstructed two chronograms of plethodontids which included representatives of: (1) all 100 taxa for ancestral life history analysis; and (2) the 63 taxa for which we have data on timing of metamorphosis and maturation. The chronograms were each based on complete datasets of 1,033 bp of the recombination activating gene 1 (*Rag1*; Additional file 1). *Rag1* was chosen because it is a conserved nuclear locus that was already available for most taxa included in this study, and provides a close approximation to the topologies and branch lengths of previously reconstructed salamander phylogenies [25,28,34,39-43]. The sequences were primarily derived from previous phylogenetic datasets of plethodontids [28], spelerpines [25], *Plethodon*[44], and additional sequences for nine species from the genus *Desmognathus* that we collected for this study (Additional file 2).

Sequences were aligned using Sequencher v. 4.8 (Gene Codes, Ann Arbor, MI, USA), and the alignment was unambiguous with no missing data. MrModeltest v. 2.2 [45] was used to determine the most appropriate model of nucleotide substitution for each codon position (Additional file 3). The chronograms were estimated using BEAST v. 1.6 [46]. We applied the best-fitting models determined above, and the analysis was based on an uncorrelated lognormal molecular clock and Yule speciation prior across the tree. The fossil record of plethodontids is very limited [47,48], so we used the base of the crown group of extant plethodontids as a calibration point. The estimates of the deepest divergence for this clade are in the range of 41 Mya to 99 Mya, with average estimates at approximately 73 Mya [40,41,49-51]. We applied a normally distributed calibration prior for the crown group of plethodontids, with a mean of 73 Mya and standard deviation of 6 Mya. This combination of parameters yielded a 95% prior distribution between 85 Mya and 65 Mya, representing a reasonable range of potential dates for this clade based on previous studies. Analyses are based on relative branch lengths of the chronograms, and would be the same regardless of the overall time scale. Both analyses were run twice independently for 20 million generations with trees saved every 1,000 generations (total 40,000 trees). Likelihood values across generations were evaluated in Tracer v. 1.5 [52] and the first 25% of generations from both runs (10,000 trees) were conservatively discarded as burnin, which was well beyond stationarity. Both chronograms (100 taxa and 63 taxa) were similar in branch lengths and topology. We used the 30,000 post-burnin trees, from the phylogenetic analysis of each dataset, for their respective reconstructions (see below).

### Ancestral state reconstruction

Ancestral life histories, ages of minimum metamorphosis, and ages of minimum maturation (males and females) of plethodontid salamanders were reconstructed using Bayesian methods. Categorical and continuous ancestral reconstructions were performed in BayesTraits v. 2.0 [53] using ‘BayesMulitState’ [54] and a Markov Chain Monte Carlo (MCMC) model. Reconstructions were based on all 30,000 post-burnin Bayesian chronograms from the phylogenetic analysis in BEAST. Uniform priors from 0 to 100 were applied for each analysis, and acceptance rates were between 20% and 40%. Each analysis was run for 5 million generations with samples taken every 1,000 generations, with the first 1 million generations of each run discarded as burnin (that is, ancestral state results were based on 4 million post-burnin generations = 4,000 samples).

Life history was reconstructed as an ordered, categorical trait with three states (direct development, biphasic, paedomorphic). Age-based traits (metamorphosis and maturation) were analyzed using both a ‘continuous’ (number of months) and categorical coding (number of years). The categorical analyses allowed for testing among alternative states for some key ancestral nodes (for example, the age of metamorphosis and maturation for the clade Spelerpini; described below). We divided continuous ages into four metamorphic age categories: 1 = 11 months or less; 2 = 12 to 23 months; 3 = 24 to 35 months; 4 = 36 months or more. A similar strategy was applied for both minimum male and female maturation, but included two additional age categories: 1 = 11 months or less; 2 = 12 to 23 months; 3 = 24 to 35 months; 4 = 36 to 47 months; 5 = 48 to 59 months; 6 = 60 months or more.

For life history reconstructions, transitions were only allowed between biphasic and direct development or biphasic and paedomorphic, but not between direct development and paedomorphic (transitions set to zero probability). Likewise, transitions between categorical age states were also ordered numerically by setting non-numerically adjacent categories to zero probability. For example, for the four metamorphic age categories, transitions were allowed in both directions between categories 1 and 2, 2 and 3, and 3 and 4, but not between 1 and 3, 1 and 4, or 2 and 4. The same strategy was applied to the six maturation age categories for males and females. Implementing ordered categories enforces ancestors to sequentially evolve through age categories (without skipping), and it also reduces the number of possible transitions for our reconstructions. Our analyses with ordered age categories were always a better fit than analyses with unrestricted transitions between categories.

All ordered transitions between states (within traits) were set to equal rates (that is, one-rate models). For each trait we compared the fit of a one-rate model to a model where transition rates (for ordered states) were allowed to vary (multi-rate models). The lowest AIC score indicates the best fitting model. ∆AIC values <3 were considered to be negligible differences between models, values ≥3 were considered moderately strong, and values ≥10 were considered very strong support for rejecting the alternative model with the higher AIC score [55]. For each trait, a one-rate model was a substantially better fit than the multi-rate model (Life history ΔAIC = 21.62; Metamorphic Age ΔAIC = 8.59; Male Maturation Age ΔAIC = 9.12; Female Maturation Age ΔAIC = 27.08).

We used BayesTraits to test for differences among ancestral conditions for key nodes in the phylogeny of spelerpines. These analyses were performed by fixing (‘fossilizing’) nodes to alternative states and comparing harmonic means (hm) for each run by calculating differences in Log Bayes Factors (LBf). The lowest LBf indicates the best fitting model [53,54]. LBf values <3 were considered negligible differences between models, values ≥3 show were considered moderately strong, and values ≥10 were considered very strong support for rejecting the alternative model with the higher LBf.

Continuous trait analyses of the timing of metamorphosis, and male and female maturation were performed in BayesTraits [53,54] using MCMC under a Brownian Motion (‘Continuous Random Walk’) model. Reconstructions were based on all 30,000 post-burnin Bayesian chronograms from the phylogenetic analysis in BEAST. Run generation parameters were the same as described above for multistate analyses, and results were based on 4,000 post-burnin samples.

**Figure 2:**
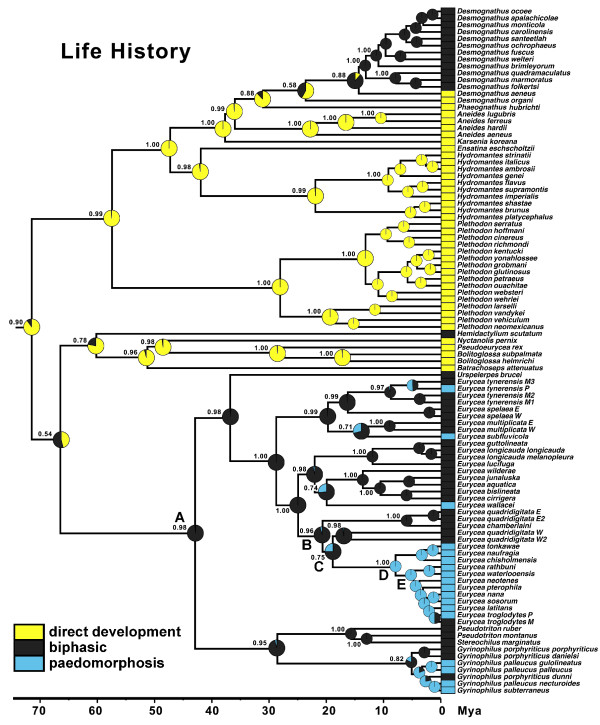
**Bayesian reconstruction of ancestral life history modes of plethodontid salamanders.** Three ordered alternative life history states are considered: direct development (yellow), biphasic (dark grey), and paedomorphic (blue). Bayesian ancestral state reconstructions were performed in BayesTraits (see Methods). Pie diagrams at each node show the proportional probability (prob.) of each state, and the highest probability subtends each node. The phylogeny is based on Bayesian analysis of *Rag1* sequences in BEAST. See also Additional file 4.

We used the Bayesian 95% Highest Prior Density (HPD) credibility interval of the ancestral state of spelerpines to determine which taxa have metamorphic ages that arose from acceleration (less than the 95% HPD interval), deceleration (greater than the 95% HPD interval), or stasis (within the 95% HPD interval; in other words, showing a lower probability of being different than our ancestral state estimate). Additionally, to test if larval form paedomorphosis arose from neoteny or progenesis, we examined timing of maturation across the evolutionary shift from metamorphosis to paedomorphosis in a large clade of paedomorphic *Eurycea* from the Edwards Plateau of Central Texas (Figure 2; node D). If paedomorphosis arose via progenesis (early maturation [1,26]), we would expect a significant reduction in maturation time concomitant with the evolution of paedomorphosis. In contrast, if paedomorphosis arose from neoteny (delayed somatic development [1,26]) then we would not expect significant differences in ancestral maturation patterns during the transition from metamorphosis to paedomorphosis. We quantified significant changes in ancestral maturation (for males and females separately using both categorical and continuous analyses). For the categorical analyses we fixed the ancestral states at four nodes spanning the evolution of paedomorphosis in Edwards Plateau *Eurycea* (Figures 2; nodes B to E) to the six alternative maturation categories (years). For a given node we used Log Bayes factors to compare which of the maturation category is the best fit, and which categories were significantly worse (methods described above). Again, for progenesis we would expect that the best fitting maturation ages would shift to younger age categories across these nodes, whereas neoteny should show no change (or an increase) in maturation age categories. We further compared the 95% HPD interval of continuous maturation age reconstructions from BayesTraits (above) for these nodes (B to E) to evaluate potential reductions in maturation time (progenesis). Male and female maturation was analyzed separately for both categorical and continuous methods.

## Results

### Reconstructions of life history mode

Reconstruction of the ancestral life history mode of plethodontids shows strong support for a direct developing ancestor for the family (prob. = 0.90; Figure 2). Fixing the common ancestor of plethodontids to direct development is a substantially better fit than biphasic (LBf = 5.05) or paedomorphic (LBf = 16.15). Inclusion of outgroup families (Amphiumidae and Rhyacotritonidae) also strongly supports a direct developing ancestor for plethodontids (prob. = 0.89; Additional file 4). Among salamanders, direct development is unique to plethodontids, and biphasic is likely ancestral for salamanders [22,23]. Previous analyses have shown that *Desmognathus* with biphasic life histories are phylogenetically nested among direct developing taxa, so free living larval stages of this clade likely result from a reversal in developmental timing [28]. Our reconstructions show that biphasic life histories in spelerpines and *Hemidactylium* are also reversals to a free-living larval stage (Figure 2). Therefore, our reconstruction supports at least three independent reversals from direct development to a biphasic life history. It should be noted that some direct developing species retain ancestral larval anatomy during development (such as larval epibranchial cartilages) [34], so we can only consider these reversals in developmental timing, or reversals to a free living larval stage, but not necessarily reversals in larval anatomy.

Similar to recent analyses [25], biphasic (metamorphic) is the ancestral state for spelerpines (prob. = 0.98; Figure 2; Table 1; Additional file 4), and there have been multiple independent instances of paedomorphosis in this clade. One important transition for our analyses of heterochronic patterns is the transition from metamorphosis to paedomorphosis in *Eurycea* from the Edwards Plateau of central Texas (taxa descending from node D; Figure 2). We find that this transition most likely occurred between node C (biphasic prob. 0.75) and node D (paedomorphic prob. = 1.00). The biphasic to paedomorphic transition along this branch (between nodes C and D) is further supported by fixing these nodes to each of the alternative life history states (Table 1). For node C, biphasic is a substantially better fit than paedomorphic (LBf = 10.88) and direct development (LBf = 22.55). In contrast, paedomorphic is a substantially better fit for the life history of node D, compared to biphasic (LBf = 9.23) or direct development (LBf = 31.26).

**Table 1:** Tests of alternative ancestral life history modes for select plethodontid nodes

**Node / Life history**	**Prob.**	**hm**	**LBf**
**Root: Plethodontidae**			
Direct development	0.90	−44.29	0.00
Biphasic	0.10	−46.82	5.05
Paedomorphic	0.00	−52.36	16.15
**Node A: Spelerpini**			
Direct development	0.01	−51.65	15.35
Biphasic	0.98	−43.98	0.00
Paedomorphic	0.01	−51.52	15.09
**Node B: *Eurycea quadridigitata*** **+ Edwards Plateau *Eurycea***			
Direct development	0.00	−55.13	21.74
Biphasic	0.96	−44.26	0.00
Paedomorphic	0.04	−51.07	13.65
**Node C: western *Eurycea quadridigitata*** **+ Edwards Plateau *Eurycea***			
Direct development	0.00	−55.49	22.55
Biphasic	0.75	−44.22	0.00
Paedomorphic	0.25	−49.65	10.86
**Node D: Edwards Plateau *Eurycea***			
Direct development	0.00	−59.97	31.26
Biphasic	0.00	−48.95	9.23
Paedomorphic	1.00	−44.33	0.00
**Node E: southern Edwards Plateau *Eurycea***			
Direct development	0.00	−65.99	43.89
Biphasic	0.00	−53.00	17.92
Paedomorphic	1.00	−44.04	0.00

Proportional probabilities are from Bayesian reconstructions of three ordered life history states (direct development, biphasic, and paedomorphic; Figure 2). Model fitting comparisons were performed by fixing (‘fossilizing’) select nodes to the three alternative life history states and comparing Log Bayes Factors (LBf) to the lowest (best fitting) life history for the node.

### Reconstructions of metamorphic age

**Figure 3:**
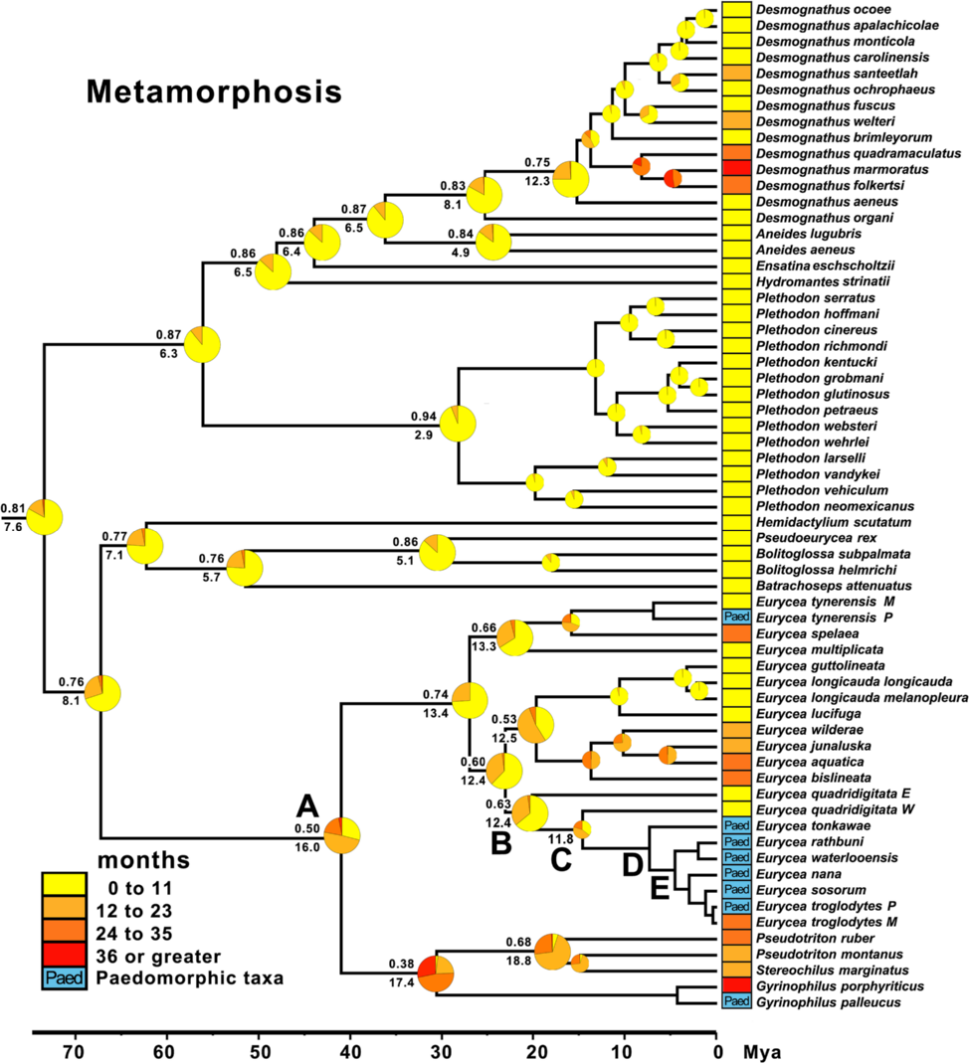
**Bayesian reconstruction of ancestral metamorphic timing in plethodontid salamanders.** Four ordered metamorphic age categories (0 to 11 months, 12 to 23 months, 24 to 35 months, and 36 months or more) were reconstructed in BayesTraits. Pie diagrams at each node show the proportional probability (prob.) of each state, and the highest probability subtends above the branch at each node. Continuous reconstruction of minimum metamorphic age under Brownian Motion was also performed in BayesTraits and the average age, from 4 million post-burnin generations, subtends each node below the branch. The phylogeny is based on Bayesian analysis of *Rag1* sequences in BEAST.

Metamorphic age is highly variable across plethodontids (Figure 3). Consistent with life history mode reconstructions (Figure 2), ancestral plethodontids also show early metamorphic age: 0 to 11 months (categorical prob. = 0.81) or 7.6 months (continuous). The 0 to 11 month age category for ancestral plethodontids (root node) is a better fit than all other age categories (Additional file 5). This estimated age of metamorphosis is within the range of hatching time for direct developers. There were two major shifts to longer metamorphic ages (Figure 3). One shift is within the genus *Desmognathus* (*quadramaculatus* species group) and the other is in the lineage leading to spelerpines. The shift in metamorphic age of spelerpines is consistent with shifts from direct development to biphasic life histories (Figure 2).

**Figure 4:**
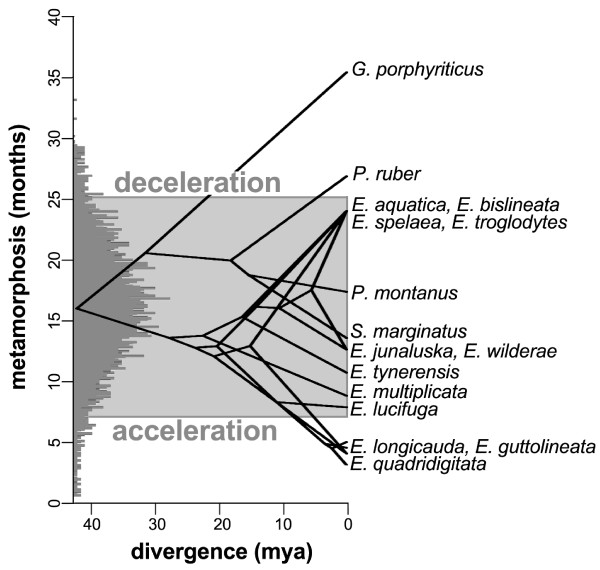
**Phenogram of metamorphic timing of spelerpine plethodontids.** The phenogram was reconstructed using phytools [56] under Brownian Motion (ancestral states are similar to those reconstructed using a Brownian Motion model in BayesTraits). The histogram is the Bayesian posterior samples from the ancestral metamorphic age estimate for spelerpines (average 16.0 months). The grey box indicates the 95% HPD interval of this estimate. Taxa above the box are considered decelerations in larval period compared to ancestral spelerpines. Taxa below the grey box are considered accelerations in larval period compared to ancestral spelerpines. Taxa within the grey box are within the range of our estimated credibility interval for the most recent common ancestor of spelerpines, and potentially represent stasis in metamorphic timing.

We estimate that ancestral spelerpines likely had a larval period of 12 to 23 months (categorical prob. 0.50) or 16.0 months (continuous). This is a better fit than older, but not younger, age categories (Additional file 5). The 95% HPD interval for our continuous metamorphic age estimate is 7.1 to 25.1 months (Figure 4; Additional file 6). Metamorphic age is highly variable among biphasic spelerpines. If we consider the 95% HPD interval for ancestral metamorphic age, then the long larval periods of *Gyrinopilus porphyriticus* (≥36 months) and *Pseudotriton ruber* (27 months; Figure 4), have a high probability of being decelerations in metamorphic timing. In comparison, *E. quadridigitata*, *E. longicauda*, and *E. guttolineata,* which minimally metamorphose in 5 months or less, have a high probability of being accelerations. All other spelerpine taxa fall within the 95% HPD interval of our ancestral state estimate, and have lower probabilities of being different from the common ancestor of spelerpines (clade A). This may reflect either undetectable shifts (using this method and criterion) or stasis in metamorphic age.

### Reconstructions of maturation age

**Figure 5:**
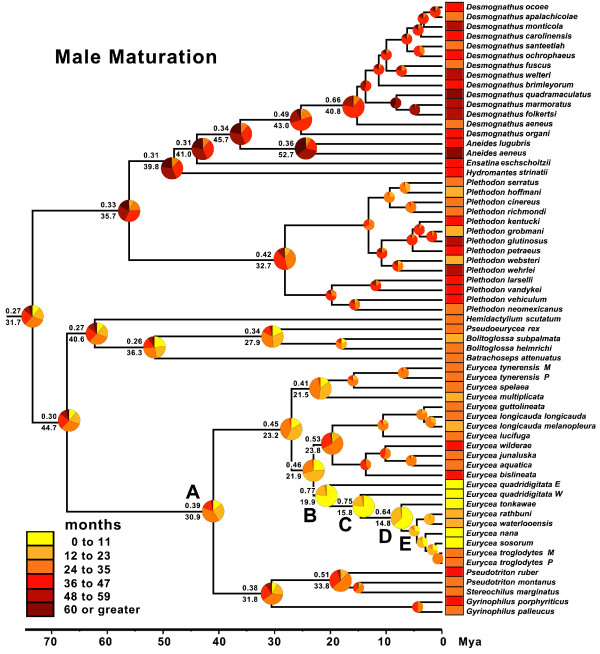
**Bayesian reconstruction of ancestral male maturation time in plethodontid salamanders.** Six ordered maturation age categories (0 to 11, 12 to 23, 24 to 35, 36 to 47, 48 to 59, and ≥60 months) for males were reconstructed in BayesTraits. Pie diagrams at each node show the proportional probability (prob.) of each state, and the highest probability subtends above the branch at each node. Continuous reconstruction of male maturation under Brownian Motion was also performed in BayesTraits and the average age, from 4 million post-burnin generations, subtends each node below the branch. The phylogeny is based on Bayesian analysis of *Rag1* sequences in BEAST.

**Figure 6:**
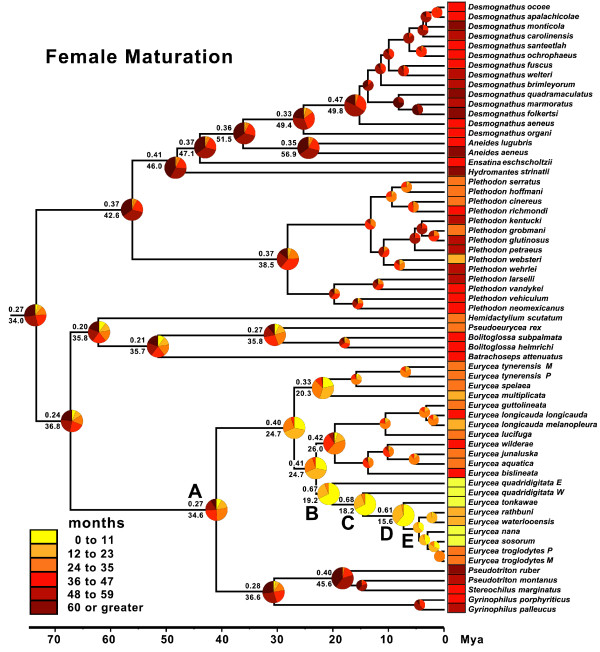
**Bayesian reconstruction of ancestral female maturation time in plethodontid salamanders.** Six ordered maturation age categories (0 to 11, 12 to 23, 24 to 35, 36 to 47, 48 to 59, and ≥60 months) for females were reconstructed in BayesTraits. Pie diagrams at each node show the proportional probability (prob.) of each state, and the highest probability subtends above the branch at each node. Continuous reconstruction of female maturation under Brownian Motion was also performed in BayesTraits and the average age, from 4 million post-burnin generations, subtends each node below the branch. The phylogeny is based on Bayesian analysis of *Rag1* sequences in BEAST.

Minimum age at maturation also varies extensively across plethodontids (Figures 5 and 6). We estimate that ancestral male spelerpines matured at 24 to 35 months (categorical prob. 0.39) or 30.9 months (continuous). Female spelerpines also likely matured at 24 to 35 months (categorical prob. 0.27) or 34.6 months (continuous). Later maturation ages are derived in *Gyrinophilus*, *Pseudotrition*, and *Stereochilus*, whereas maturation ages are reduced in some clades of *Eurycea*.

**Figure 7:**
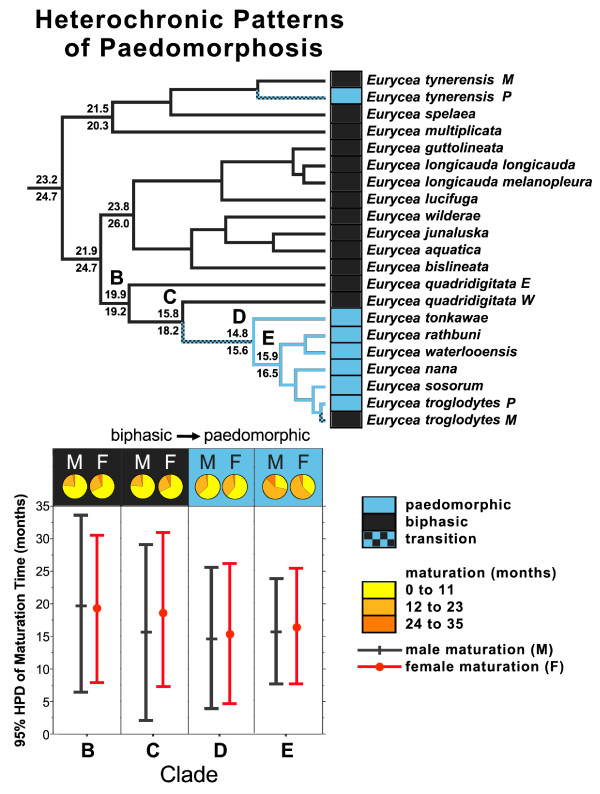
**Evolution of paedomorphosis in a clade of *Eurycea *from the Edwards Plateau.** The phylogeny shows the evolution of life history in spelerpines (biphasic *vs.* paedomorphic). The reconstruction is drawn from the ordered, three state analysis (Figure 2), and pruned to show only the taxa with maturation data (Figures 5 and 6). Four key nodes of interest are indicated (B to E), and a transitions from biphasic to paedomorphic likely occurred between nodes C and D. Proportional probabilities subtending each node are Bayesian continuous estimates of maturation time for males (above; Figure 5) and females (below; Figure 6). Pie diagrams (also extrapolated from Figures 5 and 6), show categorical estimates for nodes B to E. Vertical lines on the graph indicate the average (symbol) and 95% HPD intervals of continuous Bayesian estimates of male (black) and female (red) maturation age for the same nodes. These analyses do not provide evidence that larval form paedomorphosis in Edwards Plateau *Eurycea* (node C) evolved via progenesis, but rather indicate a case of neoteny. Major accelerations in metamorphosis and maturation likely evolved in an earlier ancestor (node B; Figures 3, 5, and 6), and early maturation age was simply maintained during the later shifts in somatic development (biphasic to paedomorphic; nodes C to D).

The earliest maturation times among plethodontids are seen in Edwards Plateau *Eurycea*, paraphyletic *E. quadridigitata*, and the common ancestors of these lineages (nodes B, C, and D). Categorical analyses support maturation ages between 0 to 11 months for males (B prob. = 0.77; C prob. = 0.75; D prob. = 0.64) and females (B prob. = 0.67; C prob. = 0.68; D prob. = 0.61). When we fixed these nodes (B to D) to alternative age categories, we find that for females the 0 to 11 age category is at least a moderately better fit than older maturation age categories (Table 2). Categorical estimates do not support a significant decrease in maturation age during the life history transition from biphasic to paedomorphic (between nodes C and D; Figure 7). Continuous estimates of maturation age for these nodes are also young, but consistently older than categorical estimates for both males (node B = 19.9; node C = 15.8; node D = 14.8; Figure 5) and females (node B = 19.2; node C = 16.2; node D = 15.6; Figure 6), with a high degree of overlap in these intervals. The estimated average maturation ages during the life history transition from biphasic to paedomorphic (between nodes C and D; Figure 7) also change very little (1 month decrease for males and 2.6 month decrease for females). Given that *E. quadridigitata* metamorphose in less than 6 months, and the estimated metamorphic ages for the common ancestors of this clade are 1 year or less (Figure 3; Additional file 6), then maturation would had to have advanced to a very early age in order to precede metamorphosis and achieve paedomorphosis via progenesis.

**Table 2:** Tests of timing of maturation for select plethodontid nodes (months)

**Node/Mat Age**	**M prob**	**M hm**	**M LBf**	**F prob**	**F hm**	**F LBf**
**Node A: Spelerpini**						
0 to 11	0.14	−106.89	10.63	0.08	−105.06	9.80
12 to 23	0.28	−104.19	5.21	0.14	−102.58	4.84
24 to 35	0.39	−101.58	0.00	0.27	−102.08	0.00
36 to 47	0.16	−104.97	6.78	0.25	−100.15	3.85
48 to 59	0.03	−105.72	8.27	0.17	−102.60	4.88
≥60	0.00	−107.79	12.43	0.09	−102.72	5.13
**Node B: *Eurycea quadridigitata*** **+ Edwards Plateau *Eurycea***						
0 to 11	0.77	−100.86	0.00	0.67	−101.08	0.00
12 to 23	0.20	−101.57	1.40	0.27	−103.66	5.15
24 to 35	0.03	−102.59	3.45	0.06	−104.03	5.90
36 to 47	0.00	−109.53	17.33	0.00	−105.84	9.52
48 to 59	0.00	−112.52	23.31	0.00	−110.84	19.52
≥60	0.00	−112.69	23.65	0.00	−112.07	21.98
**Node C: western *Eurycea quadridigitata*** **+ Edwards Plateau *Eurycea***						
0 to 11	0.75	−100.58	0.00	0.68	−99.76	0.00
12 to 23	0.22	−101.17	1.18	0.27	−101.37	3.21
24 to 35	0.03	−104.83	8.50	0.05	−104.48	9.44
36 to 47	0.00	−111.80	22.45	0.00	−108.16	16.79
48 to 59	0.00	−112.65	24.13	0.00	−111.86	24.19
≥60	0.00	−112.73	24.29	0.00	−112.35	25.16
**Node D: Edwards Plateau *Eurycea***						
0 to 11	0.64	−100.58	0.00	0.61	−99.37	0.00
12 to 23	0.35	−102.10	3.04	0.36	−103.36	7.97
24 to 35	0.01	−108.61	16.06	0.03	−106.49	14.2
36 to 47	0.00	−112.55	23.95	0.00	−111.76	24.78
48 to 59	0.00	−112.769	24.37	0.00	−112.64	26.53
≥60	0.00	−112.79	24.42	0.00	−112.74	26.74
**Node E: southern Edwards Plateau *Eurycea***						
0 to 11	0.32	−101.58	0.90	0.34	−102.28	0.77
12 to 23	0.64	−101.13	0.00	0.61	−101.89	0.00
24 to 35	0.04	−107.75	13.23	0.05	−106.87	9.95
36 to 47	0.00	−112.74	23.22	0.00	−112.44	21.09
48 to 59	0.00	−112.80	23.34	0.00	−112.78	21.77
≥60	0.00	−112.81	23.36	0.00	−112.79	21.79

Proportional probabilities (prob.) are from Bayesian reconstructions of six ordered maturation age categories (0 to 11, 12 to 23, 24 to 35, 36 to 47, 48 to 59, and ≥60 months) for males (M; Figure 5) and females (F; Figure 6). Model fitting comparisons were performed by fixing (‘fossilizing’) select nodes to the six alternative maturation age categories and comparing Log Bayes factors (LBf) to the lowest (best fitting) age category for the node based on the harmonic mean (hm).

## Discussion

### Repolarizing heterochronic shifts in plethodontids

Previous investigations of heterochronic patterns of metamorphosis and maturation for plethodontid salamanders predate robust molecular phylogenetic hypotheses [26]. Ancestral plethodontids and spelerpines were posited to be large salamanders with long larval periods and late maturation times. This led subsequent investigators to conclude that shorter larval periods of most extant metamorphic taxa were derived through multiple independent accelerations of larval development. Here we show that by reconstructing the minimum age of metamorphosis on the phylogeny of plethodontids, that ancestral spelerpines likely had relatively short, as opposed to very long, larval periods (Figure 3). When we consider the credibility interval of our estimate of ancestral metamorphic age, and the metamorphic ages of descending lineages, then it is clear that very long larval periods (for example, *G. porphyriticus* and *P. ruber*) are derived decelerations. Only a few taxa show evidence of accelerations in metamorphic timing compared to ancestral spelerpines (*E. quadridigitata*, *E. longicauda*, and *E. guttolineata*), and the rest of the taxa are either relatively unchanged since the ancestral state (stasis) or have not changed enough to detect a herterochronic shift using these methods (Figure 4).

There are multiple pathways that can lead to the evolution of larval form adult descendants (paedomorphs) from metamorphic ancestors (Figure 1). Two general mechanisms are progenesis, which acts through the acceleration (or predisplacement) of reproductive development relative to somatic development, and neoteny, which is the delay (or postdisplacement) of somatic development relative to reproductive development ([1,2,26,57]; Figure 1). Most assessments and tests of progenesis *vs.* neoteny have been performed by observing or manipulating maturation and metamorphosis within facultatively paedomorphic species [7,8,26]. However, these traits have not previously been reconstructed in a phylogenetic context to test hypotheses about origins of paedomorphosis for major lineages. Larval form paedomorphosis has evolved independently, multiple times within spelerpine plethodontids (Figure 2). Previous heterochronic interpretations, performed prior to molecular phylogenetic estimates of spelerpines, concluded that paedomorphosis evolved via delayed metamorphosis (neoteny) in *Gyrinophilus*[58], and accelerated reproduction (progenesis) in *Eurycea*[26,59,60]. A progeneic mechanism for paedomorphic evolution in *Eurycea* from the Edwards Plateau of Central Texas (our clade D) was based on the notion that this clade was most closely related to two-lined salamanders (*Eurycea bislineata* group) from the southern Appalachian Mountains, which have older maturation ages (approximately 3 years) and relatively long larval periods for a *Eurycea* (approximately 2 years). This older maturation age was assumed to have been ancestral, and therefore the younger maturation ages of Edwards Plateau *Eurycea* (for example, *E. neotenes*) were considered an acceleration of maturation relative to metamorphosis (progenesis). However, recent phylogenetic analyses show that Edwards Plateau *Eurycea* are phylogenetically nested among dwarf salamanders (*Eurycea quadridigitata*) [25,61], which have very short larval periods and maturation times more similar to Edwards Plateau *Eurycea*. Interestingly, the *Eurycea quadridigitata* group (clade B), which includes Edwards Plateau *Eurycea*, shows early maturation (progenesis) compared to other *Eurycea* and other plethodontids (Figures 5 and 6). Nevertheless, our reconstructions do not show evidence for major advancements in maturation age during the somatic shift from biphasic (metamorphic) to larval form paedomorphic development in Edwards Plateau *Eurycea* (ancestral nodes C to D; Figure 7). Therefore, even though the *Eurycea quadridigitata* group (clade B) may be generally progenic (compared to other plethodontids), we conclude that the evolution of paedomorphosis in Edwards Plateau *Eurycea* (clade D) is due to neoteny (a somatic delay of metamorphosis without a shift in maturation age). It is important to note that some studies of facultatively paedomorphic salamanders show that predisplacement (progenesis) of maturation by approximately one to two months can lead to larval form paedomorphosis [7]. The macroevolutionary methods we employed here may not be sensitive enough to detect such a subtle shift in maturation age. However, metamorphosis of *E. quadridigitata* and the common ancestors of this clade (nodes B and C) are fairly accelerated (Figures 3 and 4; Additional file 6), so maturation would have had to be considerably advanced to precede metamorphosis in this clade. More data on maturation age are needed for additional lineages of *Eurycea,* to further test if neoteny is a generalizable mechanism of larval form paedomorphosis throughout this clade or if both mechanisms can occur [8]. It is also important to perform intra and interspecific experimental comparisons to test the role of plasticity in regulating these phenomena [8] (see also below).

Our original intention for this study was to test heterochonic patterns of spelerpine plethodontids, but in doing so we also show strong support for direct development as the ancestral life history for the family (Figure 2; Additional file 4). Previous tests of this pattern showed that the radiation of biphasic desmognathines is deeply nested among direct developing taxa [28]. However, some direct developing taxa (*Hydromantes* and then undiscovered *Karsenia*[39]) were not included in that study, and some important basal relationships were still unresolved. As a consequence, the ancestral life history for plethodontids was equivocal. Our support for direct development as the ancestral life history of plethodontids (as well as other deep nodes) suggests at least two or three reversals to biphasic life histories in plethodontids (biphasic desmognathines, spelerpines, and *Hemidactylium*). It also by default suggests that direct development may have a single origin in the family. This scenario for life history evolution in the Plethodontidae has major implications for understanding morphological evolution, particularly of larval forms [62-67]. To test such questions will require individual reconstructions of morphological traits in light of new phylogenetic hypotheses and perspectives on the evolution of developmental timing in this family.

### Considering plasticity of heterochronic changes?

It is well established that environmental factors such as habitat desiccation, food availability, and temperature can influence the timing of metamorphosis and maturation in amphibians [68-71]. The potential effects of temperature on development also partly shaped previous heterochronic hypotheses [26]. This is because, in addition to the idea that ancestral plethodontids were large with long larval periods, it was also thought that the ‘center of origin’ of the family was the high elevations of the southern Appalachian Mountains [29-31,72]. This biogeographic pattern seemed consistent with the multiple independent accelerations in larval period. That is, larval development was accelerated as lineages dispersed from colder uplands to warmer lowlands. Temperature, food availability, and hydroperiod can influence the age of metamorphosis (and probably maturation) of plethodontids [9,73-77]. Nevertheless, these effects do not surpass differences among the most developmentally distinct lineages, such as the nearly 10-fold difference in minimum age of metamorphosis between *G. porphyriticus* and *E. quadridigiata*. Stark differences in metamorphic age are also found in syntopy. For example, in the southern Appalachians, age at metamorphosis varies from 12 to 24 months for *Eurycea wilderae* and 36 to 60 months for *Gyrinophilus porphyriticus*, despite the fact that these species broadly overlap in distribution and develop in the same streams (Additional file 1). Likewise, the larval periods of *E. quadridigitata* (4 to 6.5 months) and *Stereochilus marginatus* (13 to 28 months) are distinctly different, yet these species occur and develop in the same habitats on the lowland Coastal Plain.

Here we analyzed minimum metamorphic ages across species, which strongly differ among plethodontids (Figures 3 and 4), and highlights a clear pattern; that the long larval periods of *Gyrinophilus porphyriticus* and *Pseudotriton ruber* are clearly derived decelerations. However, our model for spelerpines is certainly not fixed. Environmentally induced shifts in metamorphic age could certainly push some species to accelerate or decelerate metamorphic timing compared to ancestral spelerpines, or even close relatives. For example, larval *Eurycea bislineata* in some lakes as well as far northern populations, attain a very large body sizes, and the latter are known to take at least 3 years to metamorphose [78,79]. On the opposite end of the distribution, thyroid hormone treatments of 8-month-old, lab-raised *Eurycea tynerensis* larvae (from some populations) induce significant metamorphic changes which shows that metamorphic age can be accelerated physiologically [Bonett et al., unpublished]. Further metamorphic data are needed in two important areas: (1) direct comparisons of larvae raised under common conditions to test for more subtle evolutionary shifts in heterochronic patterns among closely related species or populations; and (2) experimental tests of the degree and limits of plasticity within and among species.

### Phylogenetic analyses of heterochronic patterns

Accurate interpretations of ancestral states can have profound influences on understanding heterochronic patterns. This is because polarizing heterochronic analyses with distinctly different ancestral states can result in opposite reconstructions and interpretations of the pattern. Here we show examples of how: (1) reconstructing the ancestral timing of metamorphosis in spelerpines changes previous interpretations of patterns in the acceleration and deceleration of descending taxa (Figure 4), and 2) reconstructing maturation across a major life history transition from biphasic to paedomorphic in a clade of *Eurycea* supports a case of neoteny rather than progenesis (Figure 7).

Several methods for analyzing the evolution of heterochronic patterns have been developed over the past two decades. These methods have largely focused on ‘sequence heterochronies’, which seek to analyze the sequence of developmental changes of multiple traits among species via event-pairing (for example, [13-18,80]). Sequence heterochrony methods allow for analyses of individual elements and mixed data types, and alleviate the need for comparable data on age or size for developmental events [57]. Tracing the evolution of sequence heterochronies can also be a complex, and in some cases intractable, problem due to the high number of alternative ancestral sequences (character states) relative to the number of taxa analyzed. By comparison, ‘growth heterochrony’ (also known as deBeerian heterochrony) analyses, where size and shape are used as a proxy for age [2,3,57,81], are relatively more straightforward to reconstruct. A continuous character (for example, absolute age, relative age, size, or shape) is used to code the timing of developmental events. Data for ‘growth heterochronies’ are not always available or comparable among taxa, and therefore phylogenetic-based methods for reconstructing developmental events based on age or size have received less attention than sequence heterochrony methods. In fact, few studies have incorporated error in ancestral estimates to test for significant heterochronic shifts in a phylogenetic context [for example, 17,19].

In our study, the data available and problem at hand allow us to directly analyze the age of metamorphosis and maturation among species. We used both categorical and continuous reconstructions in our analyses to directly test heterochronic changes with respect to age, and both coding schemes (and methods) have advantages and limitations. Categorical analyses facilitate explicit hypothesis tests to analyze the best fit of alternative ancestral states (that is, age categories [54]), which extends analyses beyond examining calculated probabilities of ancestral states. An obvious advantage of a continuous analysis is that variables do not have to be partitioned into categories, which can be somewhat arbitrary, and could mask shifts in ancestral states that fall within a category. We used 12-month intervals to categorize numbers of months, and our categorical and continuous results largely agreed. Also, as we show here, variation in estimation of continuous ancestral states (for example Bayesian 95% HPD intervals) can be used to assess shifts in developmental timing between nodes, or between nodes and tips. A limitation of reconstructing continuous characters is that lineages that have undergone major shifts (in the character of interest) may effect ancestral state estimation across the tree. This is because the common models used (for example, Brownian Motion and Ornstein-Uhlenbeck) are effectively averaging tip values across ancestral nodes and may not effectively account for major rate shifts within the tree [82,83]. Another challenge is that variation in ancestral state estimation, especially for deep nodes, can be broad, which may make it difficult to detect significant heterochronic shifts between ancestors and descendants [17]. Stable trait reconstruction methods are currently being developed that account for major rate shifts when estimating continuous ancestral states [84]. These methods should offer more accurate estimates of continuous traits with lower mean squared error than Brownian Motion [84], which should improve our ability to statistically detect subtle heterochronic shifts. Despite the need for further methodological improvements, we present additional methods and examples that demonstrate how ancestral state reconstruction can be employed to test patterns of heterochronic evolution.

## Conclusions

Phylogenetic-based ancestral state reconstructions of metamorphic age (using both categorical and continuous coding) show that ancestral spelerpines had relatively shorter larval periods than previously suggested. This repolarization of the ancestral condition has major implications for understanding patterns of heterochrony in descending lineages. The long larval periods in a few taxa (for example, *Gyrinophilus porphyriticus* and *Pseudotriton ruber*) were likely derived decelerations (Figures 3 and 4), rather than a reflection of the ancestral condition. Only a few species/clades with very short larval periods (for example, *Eurycea quadridigitata* and *E. longicauda*) likely underwent accelerations in larval development. In contrast, most extant spelerpines have larval periods that are not discernably different than the ancestor for this clade (possibly the result of stasis). We also show that the evolution of paedomorphosis in an endemic radiation of *Eurycea* from the Edwards Plateau of central Texas was more likely the result of neoteny. This is because our reconstructions of ancestral maturation age show little change in the timing of maturation across the major delay in somatic morphogenesis leading to larval form paedomorphosis (Figure 7). There have been multiple independent instances of paedomorphosis in spelerpines (Figure 2), and more data on maturation times are needed to test if neoteny is a generalizable mechanism for the evolution of larval form paedomorphosis in this clade. Finally, in some systems, ancestral state reconstructions may be critical for testing ancestral conditions when polarizing patterns of heterochronic evolution.

## Competing interests

The authors declare that they have no competing interests.

## Authors’ contributions

MAS, GAR, and RMB compiled the data. RMB performed analyses and drafted the manuscript. All authors contributed to the preparation of the manuscript. All authors read and approved the final manuscript.

## Supplementary Material

Additional file 1 Table of taxa, developmental data, and Genbank accession numbers. Click here for file

Additional file 2 Table of primers and DNA sequencing methods. Click here for file

Additional file 3 Table of substitution models used in phylogenetic analyses. Click here for file

Additional file 4 Categorical life history reconstructions including outgroup families. Click here for file

Additional file 5 Additional categorical tests of timing of metamorphosis. Click here for file

Additional file 6 Additional continuous reconstructions of metamorphosis and maturation. Click here for file
