# Supplementary material for: Heterochrony repolarized: a phylogenetic analysis of developmental timing in plethodontid salamanders
Source: EvoDevo. 2014 Aug 18;5:27. doi: 10.1186/2041-9139-5-27 (PMC4169133; doi:10.1186/2041-9139-5-27)
Supplement: Additional file 1 — Table of taxa, developmental data, and Genbank accession numbers. [file 2041-9139-5-27-S1.pdf]

ADDITIONAL FILE 1: Bonett et al.

Taxa, Developmental Data, and Genbank Accession Numbers.

Taxon	LH	Meta	Mat M	Mat F	References	*Rag1*
**Hemidactyliinae**						
**Batrachosepini**						
Batrachoseps attenuatus	dd	2.8	30	36	S1-S4	JF449382
**Bolitoglossini**						
Bolitoglossa rostrata (*helmrichi*)	dd	5.5	30	42	S1,S5	AY650124
Bolitoglossa subpalmata (sp.)	dd	6.8	18	36	S1,S6	EU275810
Nyctanolis pernix	dd	-	-	-	S1	AY691714
Pseudoeurycea brunnata (*rex*)	dd	2.5	24	30	S1,S7	AY650125
**Hemidactyliini**						
Hemidactylium scutatum	bi	2.5	24	24	S1,S8-S13	AY691711
**Spelerpini**						
Eurycea aquatica	bi	24	24	24	S1,S13,S14	KF562645
Eurycea bislineata	bi	24	36	36	S1,S13,S15-S19	AY691606
Eurycea chamberlaini	bi	-	-	-	S1	KF562646
Eurycea chisholmensis	pd	-	-	-	S1	KF562647
Eurycea cirrigera	bi	-	-	-	S1,S13	KF562648
Eurycea guttolineata	bi	5	24	24	S1,S13,S21-S23	KF562651
Eurycea junaluska	bi	12	24	24	S1,S13,S24-S26	FJ750246
Eurycea latitans	pd	-	-	-	S1	KF562652
Eurycea l. longicauda	bi	5	29	36	S1,S13,S27	AY528403
Eurycea l. melanopleura	bi	5	16	16	S1,S13,S28	KF562653
Eurycea lucifuga	bi	7	30	30	S1,S13,S29-S31	KF562654
Eurycea multiplicata E	bi	-	-	-	S1	AY691707
Eurycea multiplicata W	bi	8	12	12	S1,RMB & MAS pers. obs.	KF562655
Eurycea nana	pd	-	8.5	8.5	S1,S32,JF pers. com.	KF562656
Eurycea neotenes	pd	-	-	-	S1	AY650122
Eurycea pterophila	pd	-	-	-	S1	KF562658
Eurycea quadridigitata E	bi	3	8	8	S1,S13,S33	KF562662
Eurycea quadridigitata E2	bi	-	-	-	S1	KF562659
Eurycea quadridigitata W	bi	3	8	8	S1,SET pers. com.	KF562660
Eurycea quadridigitata W2	bi	-	-	-	S1	AY691708
Eurycea rathbuni	pd	-	23	23	S1,JF pers. com.	KF562663
Eurycea sosorum	pd	-	11	11	S1,LD & DAC pers. com.	KF562664
Eurycea spelaea E	bi	-	-	-	S1	KF562666
Eurycea spelaea W	bi	24	24	24	S1,S13,S34	KF562667
Eurycea subfluvicola	pd	-	-	-	S1	KJ372324
Eurycea tonkawae	pd	-	10	10	S1,NFB & AGG pers. com.	KF562668

Eurycea tridentifera	pd	-	-	-	S1	KF562669
Eurycea troglodytes P	pd	-	24	24	S1,S13,S35	KF562670
Eurycea troglodytes M	bi	24	24	24	S1,S13,S36	KF562672
Eurycea tynerensis P	pd	-	24	24	S1,RMB & MAS pers. obs.	KF562675
Eurycea tynerensis M1	bi	-	-	-	S1	KF562673
Eurycea tynerensis M2	bi	-	-	-	S1	KF562677
Eurycea tynerensis M3	bi	10	24	24	S1,RMB & MAS pers. obs.	KF562676
Eurycea wallacei	pd	-	-	-	S1	KF562693
Eurycea waterlooensis	pd	-	18	18	S1,LD & DAC pers. com.	KF562679
Eurycea wilderae	bi	12	36	36	S1,S13,S24,S37,S38	KF562680
Gyrinophilus gulolineatus	pd	-	-	-	S1	KF562681
Gyrinophilus p. palleucus	pd	-	24	48	S1,S13,S39	KF562684
Gyrinophilus p. necturoides	pd	-	-	-	S1	KF562682
Gyrinophilus p. danielsi	bi	36	36	36	S1,S13,S40,S41	KF562687
Gyrinophilus p. dunni	bi	-	-	-	S1	KF562688
Gyrinophilus p. porphyriticus	bi	-	-	-	S1	KF562689
Gyrinophilus subterraneus	pd	-	-	-	S1	KF562692
Pseudotriton montanus	bi	17	26	48	S1,S13,S42,S43	KF562696
Pseudotriton ruber	bi	27	45	60	S1,S13,S44,S45	KF562700
Stereochilus marginatus	bi	13	33	37	S1,S13,S43,S46	AY691713
Ursplerpes brucei	bi	-	-	-	S1	KF562703
**Plethodontinae**						
**Aneidini**						
Aneides aeneus	dd	2.8	90	96	S1,S47	AY691701
Aneides ferreus	dd	-	-	-	S1	EU275805
Aneides hardii	dd	-	-	-	S1	KC165585
Aneides lugubris	dd	3	36	36	S1,S48	AY650118
**Desmognathini**						
Desmognathus aeneus	dd	1.5	24	24	S1,S49,S50	AY691696
Desmognathus apalachicolae	bi	9	24	36	S1,S50,S51	KM107911
Desmognathus brimleyorum	bi	9	36	48	S1,S50,S52	AY691697
Desmognathus carolinensis	bi	4	42	48	S1,S50,S53,S54	KM107912
Desmognathus folkertsi	bi	24	48	60	S1,S55,S56	KM107913
Desmognathus fuscus	bi	7	24	36	S1,S50	KM107914
Desmognathus marmoratus	bi	36	48	48	S1,S50,S57,S58	KM107915
Desmognathus monticola	bi	8	48	60	S1,S50,S59-S61	AY691698
Desmognathus ochrophaeus	bi	4	36	36	S1,S50,S62	KM107916
Desmognathus ocoee	bi	9	36	36	S1,S50,S60,S63	KM107917
Desmognathus organi	dd	2.5	42	42	S1,S50,S64	AY691699
Desmognathus quadramaculatus	bi	24	72	84	S1,S50,S58,S65	AY650117
Desmognathus santeetlah	bi	12	24	36	S1,S50,S66	KM107918

Desmognathus welteri	bi	20	50	50	S1,S50,S67,S68	KM107919
Phaeognathus hubrichti	dd	-	-	-	S1	AY691700
**Ensatinini**						
Ensatina eschscholtzii	dd	5	36	36	S1,S69	AY691702
**Hydromantini**						
Hydromantes ambrosii	dd	-	-	-	S1	FJ602320
Hydromantes brunus	dd	-	-	-	S1	HM797658
Hydromantes flavus	dd	-	-	-	S1	FJ602327
Hydromantes genei	dd	-	-	-	S1	FJ602340
Hydromantes imperealis	dd	-	-	-	S1	FJ602359
Hydromantes italicus	dd	-	-	-	S1	FJ602363
Hydromantes platycephalus	dd	-	-	-	S1	HM797670
Hydromantes shastae	dd	-	-	-	S1	HM797671
Hydromantes strinatii	dd	9	42	60	S70	FJ602368
Hydromantes supramontis	dd	-	-	-	S1	FJ602377
Karsenia koreana	dd	-	-	-	S1	AY887135
**Plethodontini**						
Plethodon cinereus	dd	2	24	24	S1,S71	AY691703
Plethodon glutinosus	dd	2	48	48	S1,S72	DQ995027
Plethodon grobmani	dd	2	24	24	S1,S72	DQ995028
Plethodon hoffmani	dd	2	18	28	S1,S73	DQ995029
Plethodon kentucki	dd	2	36	48	S1,S74	DQ995033
Plethodon larselli	dd	2	36	48	S1,S75	DQ995036
Plethodon neomexicanus	dd	2	24	36	S1,S76	DQ995044
Plethodon ouachitae	dd	-	-	-	S1	AY691704
Plethodon petraeus	dd	2	36	48	S1,S77	DQ995049
Plethodon richmondi	dd	2	36	48	S1,S78	DQ995051
Plethodon serratus	dd	2	24	24	S1,S79	AY691715
Plethodon vandykei	dd	2	36	42	S1,S80	AY691715
Plethodon vehiculum	dd	2	36	36	S1,S81	AY691716
Plethodon websteri	dd	2	21	21	S1,S82	DQ995073
Plethodon wehrlei	dd	2	48	48	S1,S83	DQ995075
Plethodon yonahlossee	dd	-	-	-	S1	AY691718

LH = Life History; dd = direct development; bi = biphasic; pd = paedomorphic;
Meta. = metamorphosis; Mat. = maturation. M = Male; F = Female; Ages are in months.
AGG = Andrew G. Gluesenkamp, JF = Joe Fries, LD = Laurie Dries, MAS = Michael A. Steffen,
NKB = Nathan K. Bendik, RMB = Ronald M. Bonett, SET = Stanley E. Trauth,
DAC = Dee Ann Chamberlain

Supplemental References

S1. **AmphibiaWeb: Information on amphibian biology and conservation.** [http://amphibiaweb.org/]. Berkeley, California. 2014.

S2. Hendrickson JR: **Ecology and systematics of salamanders on the genus *Batrachoseps***. *Univ Calif Publ Zool* 1954, **54**:1–46.

S3. Maiorana VC. **Size and environmental predictability for salamanders**. *Evolution* 1976, **30**:599-613.

S4. Jockusch EL, Mahoney MJ: **Communal oviposition and lack of parental care in *Batrachoseps nigriventris* (Caudata: Plethodontidae) with a discussion of the evolution of breeding behavior in plethodontid salamanders.** *Copeia* 1997, **1997**:697–705.

S5. Houck LD: **Reproductive biology of a neotropical salamander, *Bolitoglossa rostrata***. *Copeia* 1977, **1977**:70–83.

S6. Houck LD: **Growth rates and age at maturity for the Plethodontid salamander *Bolitoglossa subpalmata*.** *Copeia* 1982, **1982**:474–478.

S7. Houck LD: **Reproductive patterns in neotropical salamanders.** Dissertation 1977. University of California Berkeley.

S8. Berger-Bishop LE, Harris RN: **A study of caudal allometry in the salamander *Hemidactylium scutatum* (Caudata: Plethodontidae).** *Herpetologica* 1996, **52**:515–525.

S9. Blanchard FN: **The life history of the four-toed salamander.** *Am Nat* 1923, **57**:262–268.

S10. Blanchard FN, Blanchard FC: **Size groups and their characteristics in the salamander *Hemidactylium scutatum* (Schlegel).** *Am Nat* 1931, **65**:149–164.

S11. O'Laughlin BE, Harris RN: **Models of metamorphic timing: an experimental evaluation with the pond-dwelling salamander *Hemidactylium scutatum* (Caudata: Plethodontidae).** *Oecologia* 2000, **124**:343–350.

S12. Wood JT: **The nesting of the four-toed salamander, *Hemidactylium scutatum* (Schlegel), in Virginia.** *Am Midl Nat* 1955, **53**:381–389.

S13. Ryan TJ, Bruce RC: **Life history evolution and adaptive radiation of hemidactyliine salamanders.** In *The biology of plethodontid salamanders.* Edited by: Bruce RC, Jaeger RG, Houck LD. New York: Kluwer Academic, Plenum Publishers; 2000:303–325.

S14. Rose FL, Bush FM: **A new species of *Eurycea* (Amphibia: Caudata) from the southeastern United States**. Tulane Stud. Zool. 1963, **10**:121–128.

S15. Trapido H, Clausen RT: **The larvae of *Eurycea bislineata major*.** *Copeia* 1940, **1940**:244–246.

S16. Hudson RG: **Observations on the larvae of the salamander *Eurycea bislineata bislineata***. *Herpetologica* 1955, **11**:202–204.

S17. Stewart MM. **Population dynamics of *Eurycea bislineata* in New York.** *J Herpetol* 1968, 2:176–177.Sever DM. *Eurycea bislineata*. pp. 683.1–683.5. 1999. Catalogue of American Amphibians and Reptiles. Society for the Study of Amphibians and Reptiles, St. Louis, Missouri.

S18. Bahret R: **Ecology of lake dwelling *Eurycea bislineata* in the Shawangunk Mountains, New York.** *J Herpetol* 1996, **30**:399–401.

S19. Sever DM: ***Eurycea bislineata*.** pp. 683.1–683.5. 1999. Catalogue of American Amphibians and Reptiles. Society for the Study of Amphibians and Reptiles, St. Louis, Missouri.
S20. Gordon RE: **A population of Holbrook's salamander, *Eurycea longicauda guttolineata* (Holbrook).** *Tulane Stud Zool* 1953, **1**:55–60.
S21. Bruce RC: **The larval life of the three-lined salamander, *Eurycea longicauda guttolineata***. *Copeia* 1970, **1970**:776–779.
S22. Marshall JL: **The life-history traits of *Eurycea guttolineata* (Caudata, Plethodontidae), with implications for life-history evolution.** *Alytes* 1999, **16**:97–110.
S23. Freeman SL, Bruce RC: **Larval period and metamorphosis of the three-lined salamander, *Eurycea guttolineata* (Amphibia: Plethodontidae), in the Chattooga River watershed.** *Am Midl Nat* 2001, **145**:194–200.
S24. Bruce RC: **Egg-laying, larval periods and metamorphosis of *Eurycea bislineata* and *E. junaluska* at Santeetlah Creek, North Carolina**. *Copeia* 1982, **1982**:755–762.
S25. Sever DM: **Observations on the distribution and reproduction of the salamander *Eurycea junaluska* in Tennessee. *J Tenn Acad Sci* 1983, 58**:48–50.
S26. Ryan TJ: **Larval life history and abundance of a rare salamander, *Eurycea junaluska.*** *J Herpetol* 1998, **32**:10–17.
S27. Anderson JD, Martino PJ: **The life history of *Eurycea l. longicauda* associated with ponds.** *Am Midl Nat* 1966, **75**:257–279.
S28. Ireland PH: **Reproduction and larval development of the dark-sided salamander, *Eurycea longicauda melanopleura***. *Herpetologica* 1974, **30**:338–343.
S29. Banta AM, McAtee WL: **The life history of the cave salamander, *Spelerpes maculicaudus* (Cope).** *Proc US Nat Mus* 1906, **1443**:67–83.
S30. Hutchison VH: **Notes on the plethodontid salamanders, *Eurycea lucifuga* (Rafinesque) and *Eurycea longicauda longicauda* (Green).** *Occasional Papers of the National Spelerological Society* 1956, **3**:1–24.
S31. Clergue-Gazeau M, Thorn R: ***Eurycea lucifuga* urodela plethodontidae reproduction and development under artificial breeding conditions.** *Annales de Speleologie* 1976, **31**:169–174.
S32. Tupa DD, Davis WK: **Populations dynamics of the San Marcos Salamander, *Eurycea nana* Bishop.** *Tex J Sci* 1976, **27**:179–195.
S33. Semlitsch RD: **Growth and metamorphosis of larval dwarf salamanders (*Eurycea quadradigitata*).** *Herpetologica* 1980, **36**:138–140.
S34. Fenolio DB, Niemiller ML, Bonett RM, Graening GO, Collier BA, Stout JF: **Life history, demography, and the influence of cave-roosting bats on a population of the Grotto Salamander (*Eurycea spelaea*) from the Ozark Plateau of Oklahoma (Caudata: Plethodontidae).** *Herp Con Biol* in press.
S35. Bruce RC: **Population structure, life history and evolution of paedogenesis in the salamander *Eurycea neotenes*.** *Copeia* 1976, **1976**:242–249.

S36. Sweet SS: **Natural metamorphosis in *Eurycea neotenes*, and the generic allocation of the Texas *Eurycea* (Amphibia: Plethodontidae).** *Herpetologica* 1977, **33**:364-375.
S37. Bruce RC: **Larval periods and metamorphosis in two species of salamanders of the genus *Eurycea*.** *Copeia* 1982, **1982**:117–127.
S38. Bruce RC: **An ecological life table for the salamander *Eurycea wilderae*.** *Copeia* 1988, **1988**:15–26.
S39. Dent JN, Kirby-Smith JS: **Metamorphic Physiology and Morphology of the Cave Salamander *Gyrinophilus palleucus***. *Copeia* 1963, **1963**:119–130.
S40. Bruce RC: **Variation in the life cycle of the salamander *Gyrinophilus porphyriticus*.** *Herpetologica* 1972, **28**:230–245.
S41. Bruce RC: **Life-history patterns of the salamander *Gyrinophilus porphyriticus* in the Cowee Mountains, North Carolina.** *Herpetologica* 1978, **34**:53–64.
S42. Bruce RC: **Reproductive biology of the mud salamander, *Pseudotriton montanus*, in Western South Carolina**. *Copeia* 1975, **1975**:129-137.
S43. Bruce RC: **Life-history aspects of *Stereochilus marginatus*, with a comparison of larval development in syntopic *S. marginatus* and *Pseudotriton montanus* (Amphibia:Plethodontidae)**. *Southeastern Nat* 2008, **7**:705-716.
S44. Bruce RC: **The larval life of the red salamander, *Pseudotriton ruber*.** *J Herpetol* 1972, **6**:43–51.
S45. Bruce RC: **Reproductive biology of the salamander *Pseudotrition ruber* in the southern Blue Ridge Mountains**. *Copeia* 1978, **1978**:417–423.
S46. Bruce RC: **Life cycle and population structure of the salamander *Stereochilus marginatus* in North Carolina**. *Copeia* 1971, **1971**:234–246.
S47. Waldron JL, Pauley TK: **Green salamander (*Aneides aeneus*) growth and age at reproductive maturity.** *J Herpetol* 2007, **41**:638–644.
S48. Lee DE, Bettaso JB, Bond ML, Bradley RW, Tietz JR, Warzybok PM: **Growth, age at maturity, and age-specific survival of the arboreal salamander.** *J Herpetol* 2012, **46**:64–71.
S49. Harrison JR: **Observations on the life history, ecology and distribution of *Desmognathus aeneus aeneus*.** *Am Midl Nat* 1967, **77**:356–370.
S50. Tilley SG, Bernardo J: **Life history evolution in plethodontid salamanders.** *Herpetologica* 1993, **49**:154–163.
S51. Means BD, Karlin AA: **A new species of *Desmognathus* from the eastern Gulf Coastal Plain.** *Herpetologica* 1989, 45:37-46.
S52. Means DB: 1975. **Evolutionary ecology studies on salamanders of the genus *Desmognathus*. Part I: Competitive exclusion along a habitat gradient between two species of salamanders (*Desmognathus*) in western Florida; Part II: Life history, growth and body size variation in populations of a streamside salamander (*Desmognathus brimleyorum*) on adjacent mountains.** Ph.D. dissertation. Florida State University, Tallahassee, Florida.
S53. Tilley SG: **Life histories and natural selection in populations of the salamander *Desmognathus ochrophaeus*.** *Ecology* 1973, **54**:3–17.
S54. Organ JA: **Studies of the local distribution, life history, and population dynamics of the salamander genus *Desmognathus* in Virginia.** *Ecological Monographs* 1961, **31**:189–220.

S55. Camp CD, Tilley SG, Austin Jr RM, Marshall JL: **A new species of black-bellied salamander (genus *Desmognathus*) from the Appalachian Mountains of Georgia**. *Herpetologica* 2002, **58**:471–484.
S56. Camp CD, Marshall JL: **Reproductive life history of *Desmognathus folkertsi* (Dwarf Black-bellied Salamander).** *Southeastern Nat* 2006, **5**:669–684.
S57. Martof BS: Some aspects of the life hstory nd ecology of the salamander ***Leurognathus marmoratus.*** *Am Midl Nat* 1962, **67**:1–35.
S58. Bruce RC: **Larval periods, population structure and the effects of stream drift in larvae of the salamanders *Desmognathus quadramaculatus* and *Leurognathus marmoratus* in a southern appalachian stream.** *Copeia* 1985, **1985**:847–854.
S59. Juterbock EJ: **Sexual dimorphism and maturity characteristics of three species of *Desmognathus* (Amphibia, Urodela, Plethodontidae).** *J Herpetol* 1978, **12**:217-230.
S60. Bruce RC: ***An explanation for differences in body size between two desmognathine salamanders.*** *Copeia* 1990, **1990**:1–9.
S61. Bruce RC, Castanet J, Francillon-Vieillot H: **Skeletochronological analysis of variation in age structure, body size, and life history in three species of desmognathine salamanders.** *Herpetologica* 2002, **58**:181–193.
S62. Keen WH, Orr LP: **Reproductive cycle, growth, and maturation of northern female *Desmognathus ochrophaeus*.** *J Herpetol* 1980, **14**:7–10.
S63. Tilley SG: **Life histories and comparative demography of two salamander populations.** *Copeia* 1980, **1980**:806–821.
S64. Organ JA: **Life history of the Pigmy Salamander, *Desmognathus wrighti*, in Virginia.** *Am Midl Nat* 1961, **66**:384–390.
S65. Bruce RC: **Life history variation in the salamander *Desmognathus quadramaculatus*.** *Herpetologica* 1988, **44**:218–227.
S66. Jones RL: **Reproductive biology of *Desmognathus fuscus* and *Desmognathus santeetlah* in the Unicoi Mountains.** *Herpetologica* 1986, **42**:323–334.
S67. Juterbock EJ: **Evidence for the recognition of specific status for *Desmognathus welteri*.** *J Herpetol* 1984, **18**:240–255.
S68. Juterbock EJ: **Sexual dimorphism and matration characteristics of three species of *Desmognathus* (Amphibia, Urodela, Plethodontidae).** *J Herpetol* 1978, **12**:217–230.
S69. Stebbins RC: **Natural history of the salamanders of the plethodontid genus *Ensatina*.** *Univ Calif Publ Zool* 1954, **54**:47–124.
S70. Salvidio S: **Life history of the european plethodontid salamander *Speleomantes ambrosii* (Amphibia, Caudata).** *Herpetol J*. 1993 **3**:55–59.
S71. Sayler A: **The reproductive ecology of the Red-Backed Salamander, *Plethodon cinereus*, in Maryland.** *Copeia* 1966, **1966**:183–193.
S72. Highton R: **Geographic variation in the life history of the Slimy Salamander.** *Copeia* 1962, **1962**:597–613.
S73. Angle JP: **The Reproductive Cycle of the Northern Ravine Salamander, *Plethodon richmondi richmondi,* in the Valley and Ridge Province of Pennsylvania and Maryland.** *J Wash Acad Sci* 1969, **59**:192–202.

S74. Marvin GA: **Life history and population characteristics of the salamander *Plethodon kentucki* with a review of *Plethodon* life histories.** *Am Midl Nat* 1996, **136**:385–400.
S75. Herrington RE, Larson JH: **Reproductive biology of the Larch Mountain Salamander (*Plethodon larselli*).** *J Herpetol* 1987, **21**:48–56.
S76. Reagan DP: **Ecology and distribution of the Jemez Mountains Salamander, *Plethodon neomexicanus*.** *Copeia* 1972, **1972**:486–492.
S77. Jensen JB, Camp CD, Marshall JL: **Ecology and life history of the Pigeon Mountain Salamander.** *Southeastern Nat* 2002, **1**:3–16.
S78. Nagel JW: **Life history of the ravine salamander (*Plethodon richmondi*) in northeastern Tennessee.** *Herpetologica* 1979, **35**:38–43.
S79. Herbeck LA, Semlitsch RD: **Life history and ecology of the Southern Redback Salamander, *Plethodon serratus*, in Missouri.** *J Herpetol* 2000, **34**:341–347.
S80. Lynch JE: **Reproductive ecology of Plethodon idahoensis**. MS Thesis 1984, University of Idaho.
S81. Peacock RL, Nussbaum RA: **Reproductive biology and population structure of the Western Red-Backed Salamander, *Plethodon vehiculum* (Cooper).** *J Herpetol* 1973, **7**:215–224.
S82. Semlitsch RD, West CA: **Aspects of the life history and ecology of Webster's Salamander, *Plethodon websteri*.** *Copeia* 1983, **1983**:339–346.
S83. Hall RJ, Stafford DP: **Studies in the life history of Wehrle's Salamander, *Plethodon wehrlei*.** *Herpetologica* 1972, **28**:300–309.
