# Supplementary material for: Heterochrony repolarized: a phylogenetic analysis of developmental timing in plethodontid salamanders
Source: EvoDevo. 2014 Aug 18;5:27. doi: 10.1186/2041-9139-5-27 (PMC4169133; doi:10.1186/2041-9139-5-27)
Supplement: Additional file 2 — Table of primers and DNA sequencing methods. [file 2041-9139-5-27-S2.pdf]

ADDITIONAL FILE 2: Bonett et al.

Primers used to for PCR and sequencing.

Gene	Primer Name	Primer sequence	Reference
Rag1	DESMOG_Rag1_F	5'–CGGCAGATATTCCAGCCTTTAC–3'	This study
	DESMOG_RAG1_R	5'–CGATGGAGCCATCTCGCTCTATGA–3'	This study
	DESMOG_RAG1_INT_F*	5'–GGGTACAGGCTATGATGAGAAG–3'	This study

* Internal primers used for sequencing only.

DNA Sequencing methods

DNA was extracted from fresh, frozen, or ethanol preserved tissues using a Qiagen DNeasy extraction kit. Specimens were handled in accordance with Institutional Animal Care and Use Committee (IACUC) protocols at the University of Tulsa (TU-0029). *Rag1* was amplified using polymerase chain reactions (PCR) with newly designed primers (above), and the following conditions:

Standard PCR Conditions (25 µl reaction):
16.0 µl - DNAse/RNAse Free Water
5.0 µl - 5x PCR Buffer (GoTaq Promega ®)
1.5 µl - 25mM MgCl2
0.5 µl - 10 µM Forward Primer
0.5 µl - 10 µM Reverse Primer
0.5 µl - 10 mM dNTPs
0.5 µl - Taq Polymerase (GoTaq Promega ®)
1.0 µl - DNA template (10 to 100 ng/µl)

Standard PCR Cycling:
#1 - 95°C - 3 minutes (initial denaturing)
#2 - 95°C - 30 seconds (denaturing)
#3 - 57 to 59°C - 30 seconds (annealing)
#4 - 72°C - 45 to 90 seconds (extension)
#5 - Cycle through steps 2 to 4 - 45 times
#6 - 72°C - 10 minutes (final extension)
#7 - 8°C (Hold)

PCR products were checked on 1% agarose gels and successful PCR products were cleaned with EXOSAPIT (USB Corp.). In instances where multiple bands were amplified, entire PCR products were run out on a 1% agarose gel and bands of the correct molecular weight were physically excised with a scalpel and extracted using a Qiagen Gel Extraction kit. Cycle sequencing was performed with Big Dye v 3.1 (Applied Biosystems Inc.). Unincorporated dye terminators were removed from sequencing reactions with Sephadex G-50 (Sigma) and sequenced on an ABI 3130xl capillary sequencer at the University of Tulsa. Sequences were aligned and edited using Sequencher v. 4.8 (Gene Codes, Ann Arbor, MI). All *Rag1* sequences were the same length, with no codon insertions or deletions, and the alignment was unambiguous.
