# Supplementary material for: Heterochrony repolarized: a phylogenetic analysis of developmental timing in plethodontid salamanders
Source: EvoDevo. 2014 Aug 18;5:27. doi: 10.1186/2041-9139-5-27 (PMC4169133; doi:10.1186/2041-9139-5-27)
Supplement: Additional file 3 — Table of substitution models used in phylogenetic analyses. [file 2041-9139-5-27-S3.pdf]

ADDITIONAL FILE 3: Bonett et al.

Models applied to each data partition for Bayesian phylogenetic analyses and divergence time estimation.

Gene/partition	# of positions in partition	Substitution model	Rates
Rag1: alignment pos 1, codon pos 1	345	GTR	gamma
Rag1: alignment pos 2, codon pos 2	344	HKY	I + gamma
Rag1: alignment pos 3, codon pos 3	344	GTR	gamma

GTR = general time reversible model. HKY = Hasegawa-Kishino-Yano model. I = proportion of invariant sites. Total fragment length for *Rag1* = 1,033 bp.
