# Supplementary material for: Heterochrony repolarized: a phylogenetic analysis of developmental timing in plethodontid salamanders
Source: EvoDevo. 2014 Aug 18;5:27. doi: 10.1186/2041-9139-5-27 (PMC4169133; doi:10.1186/2041-9139-5-27)
Supplement: Additional file 4 — Categorical life history reconstructions including outgroup families. [file 2041-9139-5-27-S4.pdf]

ADDITIONAL FILE 4: Bonett et al.

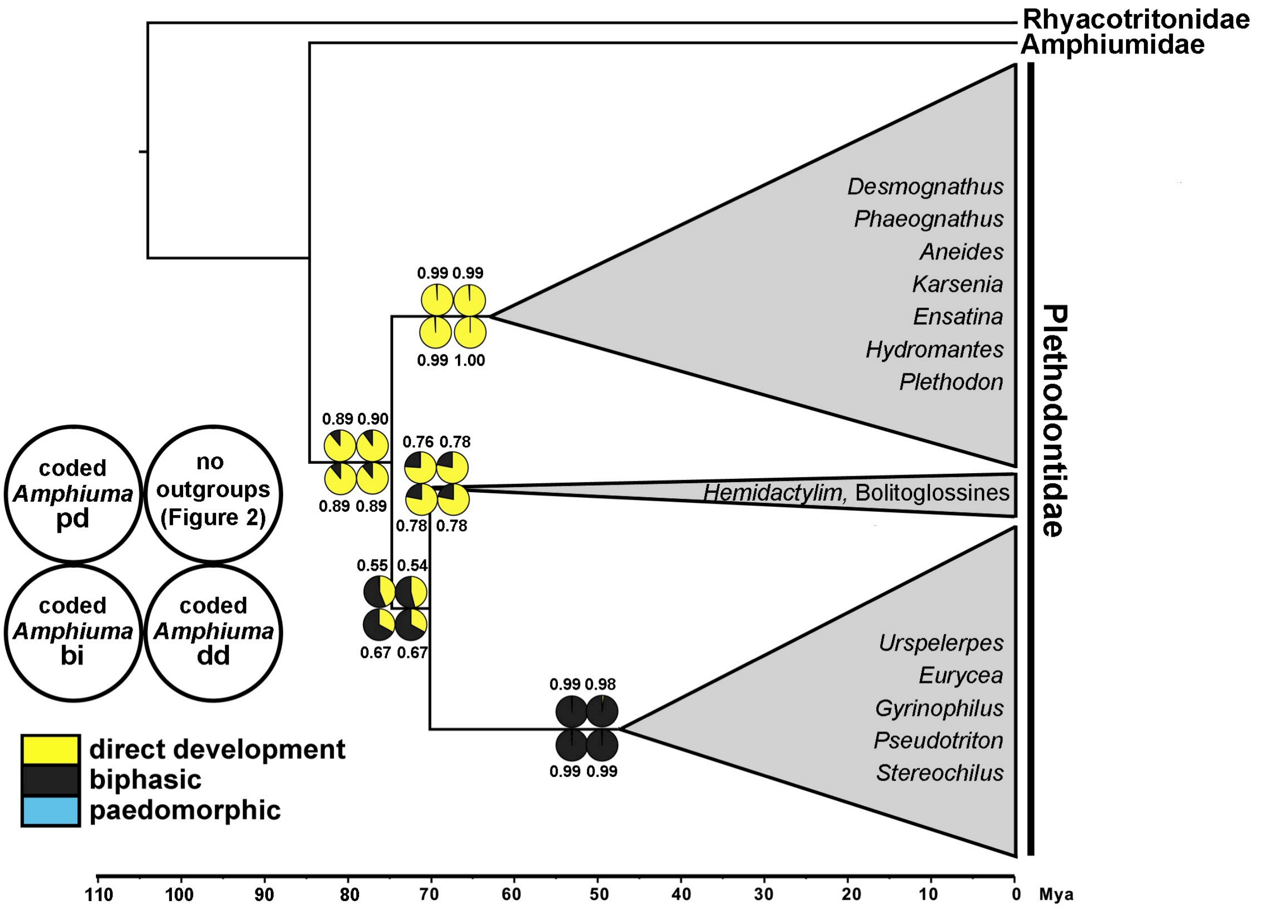

Categorical ancestral state reconstruction of plethodontid life histories including outgroups. Bayesian reconstructions were performed using the same methods as described in the main manuscript (for Figure 2) except for the inclusion of the outgroup families (Amphiumidae and Rhyacotritonidae) in the three additional analyses presented here. These families are resolved as the closest relatives to plethodontids in most molecular phylogenetic studies over the last decade. For diagrammatic purposes only, three major plethodontid clades were collapsed to simplify the phylogeny, which is intended to highlight the basal nodes within the family. Pie diagrams subtending major nodes show the proportional probability of each life history state: direct development (dd), biphasic (bi), and paedomorphic (pd). The value adjacent to the pie is the proportional probability of the most probable state (highest value). The upper right pie diagram at each node is from the analysis in Figure 2 (without outgroups). For the three analyses with outgroups, *Rhyacotriton* were always coded as biphasic, whereas *Amphiuma* were coded as direct developers (lower right pie), biphasic (lower left pie), or paedomorphic (upper left pie). Note: including these outgroups (and regardless of how *Amphiuma* was coded) had almost no effect on the ancestral life history estimates for plethodontids. Maximum Likelihood reconstructions also performed in BayesTraits yield almost identical values; with and without outgroups.
