# Supplementary material for: Heterochrony repolarized: a phylogenetic analysis of developmental timing in plethodontid salamanders
Source: EvoDevo. 2014 Aug 18;5:27. doi: 10.1186/2041-9139-5-27 (PMC4169133; doi:10.1186/2041-9139-5-27)
Supplement: Additional file 5 — Additional categorical tests of timing of metamorphosis. [file 2041-9139-5-27-S5.pdf]

ADDITIONAL FILE 5: Bonett et al.

Tests of timing of metamorphosis for select plethodontid nodes.

Node / Metamorphic Age	Probability	Harmonic Mean	Log BF
**Root: Plethodontidae**			
0 to 11 months:	0.81	-48.67	0.00
12 to 23 months:	0.17	-51.25	5.16
24 to 35 months:	0.02	-53.12	8.90
36 or greater months:	0.00	-55.72	14.10
**Node A: Spelerpini**			
0 to 11 months:	0.27	-49.60	0.18
12 to 23 months:	0.50	-49.51	0.00
24 to 35 months:	0.20	-51.58	4.14
36 or greater months:	0.03	-55.17	11.32

Proportional probabilities are from Bayesian reconstructions of four ordered metamorphic age categories (0 to 11, 12 to 23, 24 to 35, and 36 or greater months; Figure 3). Model fitting comparisons were performed by “fossilizing” select nodes to the four alternative metamorphic age categories and comparing Log Bayes Factors (LBf) to the lowest (best fitting) maturation age category for the node.
