# Supplementary material for: Heterochrony repolarized: a phylogenetic analysis of developmental timing in plethodontid salamanders
Source: EvoDevo. 2014 Aug 18;5:27. doi: 10.1186/2041-9139-5-27 (PMC4169133; doi:10.1186/2041-9139-5-27)
Supplement: Additional file 6 — Additional continuous reconstructions of metamorphosis and maturation. [file 2041-9139-5-27-S6.pdf]

ADDITIONAL FILE 6: Bonett et al.

Timing of metamorphosis and maturation for select plethodontid nodes as a continuous trait.

Node	Met average	Met 95% HPD	Male Mat average	Male Mat 95% HPD	Female Mat average	Female Mat 95% HPD
**Root:**	7.6	1.0 – 14.1	31.7	15.2 – 48.3	34.0	22.9 – 52.9
**Node A:**	16.0	7.1 – 25.1	30.9	11.9 – 51.7	34.6	17.9 – 52.3
**Node B:**	12.4	5.4 – 18.5	19.9	6.4 – 33.6	19.2	7.9 – 30.5
**Node C:**	11.8	5.5 – 19.5	15.8	2.0 – 29.1	18.2	7.2 – 30.9
**Node D:**	-	-	14.8	3.9 – 25.6	15.6	4.7 – 26.2
**Node E:**	-	-	15.9	7.7 – 23.8	16.5	7.7 – 25.4

Bayesian ancestral state reconstructions of minimum metamorphic age, minimum male maturation, and minimum female maturation at the root (Plethodontidae) and five other key nodes in the tree (Figures 3, 5, and 6). Analysis is based on continuous reconstruction under Brownian Motion in BayesTraits. Averages and 95% highest prior density for each character at each node were calculated in Tracer. Met = Metamorphosis; Mat = Maturation; hyphen (-) = nodes not present in analysis.
